# Differences in metabolic transport and resistance mechanisms of Abemaciclib, Palbociclib, and Ribociclib

**DOI:** 10.3389/fphar.2023.1212986

**Published:** 2023-07-05

**Authors:** Zhimin Zhu, Qiongni Zhu

**Affiliations:** ^1^ Department of Pharmaceutics, Shanghai Eighth People’s Hospital, Shanghai, China; ^2^ Department of Pharmacy, Ruijin Hospital, Shanghai Jiao Tong University School of Medicine, Shanghai, China

**Keywords:** targeted therapy, CDK4/6 inhibitors, metabolizer transport, drug resistance, Abemaciclib, Palbociclib, Ribociclib

## Abstract

Cyclin-dependent kinase 4/6 inhibitors (CDK4/6i) play a crucial role in cancer treatment, particularly in breast cancer, and their mechanism of drug resistance is a topic of global interest in research. Hence, it is vital to comprehend the distinctions between various CDK4/6i, including their mechanisms of action and resistance mechanisms. This article aims to summarize the metabolic and transport variations as well as the differences in resistance among the three FDA-approved CDK4/6 inhibitors: Abemaciclib, Palbociclib, and Ribociclib. It also aims to discuss how these differences impact the effectiveness and safety of anticancer drugs. It was conducted in March 2023 to search PubMed, Embase, and Web of Science for literature related to this topic. Despite all being CDK4/6i, differences in their metabolism and transport were found, which are related to their chemical structure. Moreover, there are variations in preclinical pharmacology, pharmacokinetics, and clinical safety and efficacy of the different inhibitors. Genetic mutations, drug tolerance, and other factors may influence CDK4/6 resistance mechanisms. Currently, the resistance mechanisms differences of the three drugs remain largely unknown, and there are differences in the resistance mechanisms among them, necessitating further exploration and research.

## 1 Introduction

Dysregulation of the cell cycle and persistent cell proliferation due to cyclin-dependent kinase (CDK) activation are important markers of tumorigenesis. Currently marketed CDK4/6 inhibitors (CDK4/6i) belong to the third generation of CDK inhibitors. In compared to the non-selective CDK inhibitors such as Flavopiridol, Roscovitine and Olomucine, as well as broad-spectrum CDK inhibitors including Dinaciclib and AG-024322, CDK4/6i inhibitors demonstrate the ability to impede the cell cycle from G1 to S phase and, while also maintaining a superior equilibrium between antitumor efficacy and safety (Chen et al., 2016). Until now, a total of 5 CDK4/6i have been approved for marketing worldwide, and multiple CDK4/6i are still in the clinical research. Abemaciclib, Ribociclib, Palbociclib, and Trilaciclib have been approved by the Food and Drug Administration (FDA) for marketing, and all except Trilaciclib, which is used for chemotherapy-induced bone marrow suppression, are used for cancer treatment. Dalpiciclib (SHR6390) was approved by the National Medical Products Administration (NMPA) on 31 December 2021, in China.

CDK4/6 inhibitors play a crucial role in cancer treatment, particularly in breast cancer, and their mechanism of drug resistance is a topic of global interest in research (Pang et al., 2022). The latest domestic and foreign guidelines recommend the use of CDK4/6i for adjuvant and advanced treatment of hormone receptor-positive (HR+) breast cancer patients (Gradishar et al., 2022; Li and Jiang, 2022). Even though CDK4/6i have shown significant clinical benefits in HR + breast cancer (Scheidemann and Shajahan-Haq, 2021; Xu et al., 2021). Studies indicate that approximately 20% of HR + breast cancer treated with CDK4/6i experience primary resistance (Kong et al., 2019), while over 30% of patients develop secondary resistance (O'Leary et al., 2018). The development of resistance in almost all patients during treatment poses new challenges for managing this disease (Scheidemann and Shajahan-Haq, 2021). Multi-omics analysis has revealed that distinct CDK4/6i exhibit pharmacological and clinical activity differences. Moreover, these CDK4/6i drugs induce significant variations in transcriptional, proteomic, and phenotypic changes (Hafner et al., 2019). The toxicity and adverse effects of CDK4/6i differ significantly. For instance, Abemaciclib causes gastrointestinal toxicity, Palbociclib is mainly hemotoxic, and Ribociclib increases the risk of cardiotoxicity (Gao et al., 2020). A study has demonstrated that patients who exhibit resistance to Palbociclib with a high expression of cyclin E1 (CCNE1) may derive greater benefits from treatment with Abemaciclib as opposed to Ribociclib (Turner et al., 2019). It is vital to comprehend the distinctions between various CDK4/6i, including their mechanisms of action and resistance mechanisms. This article aims to provide a summary of the metabolic and transport variations and resistance differences of the three CDK4/6i (Abemaciclib, Palbociclib, and Ribociclib) that have been approved by the FDA for treating tumors. It also aims to discuss how these differences impact the effectiveness and safety of anticancer drugs.

## 2 Metabolic and transport differences

CDK4/6i are oral medications, which makes them a convenient treatment option for patients. Metabolic and transport difference have been identified for Abemaciclib, Palbociclib, and Ribociclib in Table 1, which may influence the efficacy and safety. By understanding these genetic differences, healthcare professionals can tailor the treatment to the individual patient, potentially improving outcomes and reducing adverse effects. The three CDK4/6i are primarily metabolized in the liver via the cytochrome P4503A4 enzyme (CYP3A4) (Yu et al., 2017; Sorf et al., 2018; Yu et al., 2019). Abemaciclib is a sensitive substrate for CYP3A4 (Yu et al., 2019), which means that the activity of this enzyme can significantly impact the drug’s metabolism and elimination from the body. On the other hand, Ribociclib is a strong inhibitor of CYP3A (Sorf et al., 2018; Yu et al., 2019), which can potentially increase the concentration of other drugs that are metabolized through this pathway. It is important to note that Palbociclib is also affected by CYP3A4, but it does not appear to be a sensitive substrate or a strong inhibitor. The CYP3A4 mediated interaction can increase the risk of adverse effects, such as toxicity or drug interactions, and may require dose adjustments or avoidance of co-administration with other CYP3A substrates or inhibitors for Palbociclib (Martínez-Chávez et al., 2019; Molenaar-Kuijsten et al., 2022; Patil et al., 2022) and Ribociclib (Patil et al., 2022). A Study has shown that transgenic CYP3A4 drastically reduces the plasma concentration of Abemaciclib (Martínez-Chávez et al., 2022). However, there is also another study suggested that Abemaciclib does not influence CYP3A4 substrates pharmacokinetics which has used mild inhibitors (Turner et al., 2020). In short, the three CDK4/6i are primarily metabolized by CYP3A4 *in vivo*, but the extent of this influence may differ between the drugs. Therefore, it is important to consider potential drug interactions and the patient’s individual characteristics when selecting and dosing CDK4/6i to optimize treatment outcomes and minimize adverse effects.

**TABLE 1:** Metabolic and transport differences of Abemaciclib, Palbociclib, and Ribociclib.

Biomarkers	Abemaciclib	Palbociclib	Ribociclib
CYP3A4	Sensitive substrates (Yu et al., 2019)	Palbociclib is mainly metabolized by CYP3A4 (Yu et al., 2017)	Strong inhibitors (Sorf et al., 2018; Yu et al., 2019)
	Transgenic CYP3A4 drastically reduced the Abemaciclib plasma (Martínez-Chávez et al., 2022)	CYP3A4-mediated drug interactions with Palbociclib (Molenaar-Kuijsten et al., 2022; Patil et al., 2022)	CYP3A4-mediated drug interactions with Ribociclib (Patil et al., 2022)
	No significant impact on pharmacokinetics (Turner et al., 2020)		CYP3A4 restricts Ribociclib oral bioavailability (Martínez-Chávez et al., 2019)
CYP3A5	—	CYP3A5*1/*3 may lead to enhanced drug metabolism and reduced plasma concentration (Roncato et al., 2022)	—
CYP1A2	No significant impact on pharmacokinetics (Turner et al., 2020)	—	Ribociclib inhibits CYP1A2 (Sorf et al., 2018)
CYP2C9	No significant impact on pharmacokinetics (Turner et al., 2020)	—	Ribociclib inhibits CYP2C9 (Sorf et al., 2018)
CYP2D6	No significant impact on pharmacokinetics (Turner et al., 2020)	—	—
SULT2A1	—	Responsible for metabolizing 26% of Palbociclib (Yu et al., 2017)	—
ABCB1	ABCB1 2677G>T/A homozygous type is associated with a higher abemaciclib concentration (Maeda et al., 2022; Maeda et al., 2023)	Palbociclib resistance is mediated by ABCB1 (Fu et al., 2022)	Substrate and potent inhibitor of ABCB1 (Sorf et al., 2018)
	Remarkably enhanced the efficacy of chemotherapeutic drugs in ABCB1 or ABCG2 over-expressing cancer (Wu et al., 2017)	ABCB1_rs1128503 is potential risk factors for neutropenia (Iwata et al., 2021)	P-glycoprotein limits Ribociclib brain exposure (Martínez-Chávez et al., 2019)
	ABCB1 limit brain penetration and total plasma exposure of abemaciclib (Martínez-Chávez et al., 2022)	Palbociclib is a substrate of P-gp (de Gooijer et al., 2015)	
		ABCB1-rs1128503, rs1045642, and rs2032582 may increase oral drug absorption and plasma concentration (Roncato et al., 2022)	
ABCG2	ABCG2 limit brain penetration and total plasma exposure of abemaciclib (Martínez-Chávez et al., 2022)	Palbociclib is a substrate of BCRP (de Gooijer et al., 2015)	Ribociclib as a potent inhibitor of ABCG2 (Sorf et al., 2018)
OATP1B1	—	Fatal Statin induced rhabdomyolysis by possible interaction with Palbociclib through OATP1B1 (Nelson et al., 2017)	Ribociclib can potentially inhibit OATP1B1 (Streicher et al., 2021)
OATP1B3	—	—	Ribociclib can potentially inhibit OATP1B3 (Streicher et al., 2021)

Although CYP3A4 is the primary enzyme responsible for the metabolism of CDK4/6i, other liver enzyme metabolism genes may also play a role in drug efficacy and safety. CYP3A5 is a closely related enzyme to CYP3A4 (Hlavica, 2017). One study found that the CYP3A5 *1/*3 genotype may associated with lower plasma concentrations of Palbociclib, suggesting that individuals with the CYP3A5 1/*3 genotype may exhibit a more effective metabolism of Palbociclib (Roncato et al., 2022). Nevertheless, there is insufficient data regarding the potential influence of CYP3A5 genetic variations on the metabolism and pharmacokinetics of Abemaciclib and Ribociclib. Some studies have explored the effects of other metabolism genes on the pharmacokinetics and pharmacodynamics of CDK4/6i. One study suggested that Ribociclib may have the potential for drug-drug interactions by inhibiting CYP1A2 and CYP2C9 (Sorf et al., 2018), which are important enzymes in drug metabolism. On the other hand, another study found that Abemaciclib did not influence the pharmacokinetics of CYP1A2, CYP2C9, and CYP2D6 substrates (Turner et al., 2020). However, there is limited data available on the potential impact of metabolism genes on the pharmacokinetics and pharmacodynamics of Palbociclib. It has been reported that approximately 26% of Palbociclib is metabolized by sulfotransferase family 2A member 1 (SULT2A1) (Yu et al., 2017). However, there is currently no information available regarding the potential role of SULT2A1 in the metabolism and pharmacokinetics of Abemaciclib and Ribociclib. These findings suggest that genetic variations may influence the metabolism and response to CDK4/6i, and personalized dosing strategies may be necessary to optimize treatment outcomes. A deeper understanding of the clinical implications of genetic variability in CDK4/6i metabolism depends on further studies to confirm these findings.

There are still some differences in the metabolism and transport that have not been fully elucidated. Studies have shown that Palbociclib is a substrate for ATP Binding Cassette Subfamily B Member 1 (ABCB1, also known as P-glycoprotein) and ATP binding cassette subfamily G member 2 (ABCG2, protein BCRP) (de Gooijer et al., 2015; Martínez-Chávez et al., 2019; Fu et al., 2022). Ribociclib is a substrate of ABCB1 and a potent inhibitor of ABCB1 and ABCG2 (Sorf et al., 2018), while Abemaciclib acts as both substrate (Martínez-Chávez et al., 2022) and inhibitor (Wu et al., 2017). Therefore, Abemaciclib has been shown to be less efflux efficient than Palbociclib and penetrates the central nervous system better (Raub et al., 2015). Studies have investigated the relationship between CDK4/6i and efflux transporters. Abemaciclib concentrations are higher when ABCB1 2677G>T/A is homozygous (Maeda et al., 2022; Maeda et al., 2023). Palbociclib oral absorption and plasma levels may be affected by ABCB1-rs1128503, rs1045642, and rs2032582 (Roncato et al., 2022). ABCB1_rs1128503 is potential risk factor for Palbociclib grade 3/4 neutropenia in non-Asian patients (Iwata et al., 2021). Fatal Statin-induced rhabdomyolysis may be a consequence of the interaction with Palbociclib (Nelson et al., 2017) and Ribociclib (Streicher et al., 2021) by organic anion transporting polypeptides OATP1B1 and OATP1B3. Some studies have suggested that the differences in efflux transport of the three CDK4/6i may be related to their chemical structures (Abdelmalak et al., 2022), while others have suggested that there may be other factors involved that are not yet fully understood. Additionally, there is some controversy in the literature regarding the role of efflux transporters in the pharmacokinetics and pharmacodynamics of CDK4/6i. It is still necessary to conduct further research to fully understand the mechanisms of CDK4/6i metabolism, transport and to identify potential strategies to optimize their efficacy and safety.

## 3 Chemical structure, preclinical pharmacology, pharmacokinetic differences

The CDK4/6i have different chemical structures, preclinical pharmacology, and pharmacokinetic differences which may affect their efficacy and safety profiles (George et al., 2021). Abemaciclib, Palbociclib, and Ribociclib were derivatives of pyrimidine, pyridopyrimidine, triazolopyridine, respectively. Abemaciclib is unique among the three drugs in that it contains two fluorine atoms (Chen et al., 2016). Since Ribociclib and Palbociclib have larger substituents, they are more lipophilic and have larger binding sites than Abemaciclib (Marra and Curigliano, 2019). This can affect the drugs’ pharmacokinetics and pharmacodynamics, including their absorption, distribution, metabolism, and elimination in the body, as well as their specific interactions with target proteins. Palbociclib, Ribociclib, and Abemaciclib have slightly different selectivity for different cyclin-dependent kinases (Braal et al., 2021). A study has shown that Abemaciclib inhibits CDK4 more selectively than CDK6, with a selectivity ratio of about 5:1, which may result in lower hematological toxicity than Palbociclib and Ribociclib. However, Abemaciclib also has inhibitory effects on other kinases (Kim et al., 2018), including CDK2/Cyclin A/E, CDK4/6, and CDK1/Cyclin B which is associated with CDK resistance (Hafner et al., 2019), resulting in specific gastrointestinal toxicity of Abemaciclib. In contrast, Ribociclib has a higher selectivity for inhibiting CDK4 with weaker inhibition of other kinases, However, there are some adverse effects in the electrocardiogram. The transcriptional, proteome, and phenotypic changes induced simultaneously by Palbociclib, Ribociclib, and Abemaciclib are significantly different (Hafner et al., 2019). Palbociclib, Ribociclib, and Abemaciclib have different pharmacokinetic profiles, including their half-life. According to the drug labels and clinical studies, the different pharmacokinetic profiles of these drugs may affect their dosing schedules, frequency of administration, and potential drug interactions.

Abemaciclib is typically administered twice daily at a dosage of 150 mg, whereas Palbociclib is given once daily at a 125 mg dosage for 21 consecutive days, followed by a 7-day interval. Ribociclib, on the other hand, is prescribed at a daily dosage of 600 mg for a 3-weeks period, followed by a 1-week break, with the option of adjusting the dosage based on individual patient considerations. The varying dosing schedules may impact treatment adherence and patient quality of life (Higano and Hafron, 2023; Koni et al., 2023), as some patients may prefer less frequent dosing while others may tolerate daily dosing better. Furthermore, the expenses and resource allocation linked to the administration of these drugs may be influenced by the dosing regimens (Srivastava et al., 2013; Patel et al., 2023), particularly in outpatient environment where frequent monitoring and interventions may be required. It is important to note that while the endorsed doses and schedules for these agents founded on clinical trials and practical experience, the heterogeneity in drug exposure due to variations in metabolism and clearance among patients may lead to inter-patient differences in effectiveness and adverse effects. Consequently, the utilization of pharmacodynamic biomarkers or serum drug levels for therapeutic drug monitoring may facilitate the optimization of dosing and reduction of toxicity in specific patient cohorts. Additionally, clinical investigations have demonstrated that these medications may exhibit distinct profiles of adverse effect, necessitating the implementation of dose reductions or interruptions to mitigate toxicities. As a result, drug recommendations may differ across populations (El Rassy et al., 2018), underscoring the importance of meticulously evaluating each drug’s dosing regimen, potential side effects, and patient-specific factors to optimize the efficacy and safety of CDK4/6 inhibitor therapy. In addition, ongoing research is required to examine the most effective administration schedules, sequencing, and amalgamations of these agents, along with their enduring toxicity and efficacy profiles.

## 4 The resistance mechanisms to different CDK4/6i

The mechanism of resistance to CDK4/6i can be influenced by various factors, such as genetic mutations, drug tolerance, cellular environment, drug resistance genes, and drug metabolism. Including abnormal CDK activity, loss of PTEN function (Costa et al., 2020), AKT1 (Alves et al., 2021), PI3K mutation (Bardia et al., 2021), mTOR (Yoshida et al., 2019), CDK2 activation, etc. According to studies, Abemaciclib cross-resistance is incomplete with Palbociclib or Ribociclib. In spite of the fact that they are all CDK4/6i, their chemical structures and inhibitory profiles differ slightly from one another, which may lead to different activities and resistance mechanisms in different cells and tumor types. In this article we subdivided the different resistance mechanisms (Table 2).

**TABLE 2:** The resistance mechanisms to Abemaciclib, Palbociclib, and Ribociclib.

Biomarker	Cancer	Description	Abemaciclib	Palbociclib	Ribociclib
RB1	HR + BC	Genetic and epigenetic deactivation of RB1 gene results in resistance to CDK4/6 inhibitors; RB1 serves as a valuable marker of resistance	Yoshida et al. (2019)	O'Leary et al. (2018); Costa et al. (2020); Alves et al. (2021); Bardia et al. (2021); Yoshida et al. (2019); Malorni et al. (2016); Herrera-Abreu et al. (2016)	Alves et al. (2021); Bardia et al. (2021)
	Melanoma				
PI3K-PDK1	ER + BC	Ribociclib-resistant breast cancer cells selected by chronic drug exposure displayed a relative increase in the levels of PDK1 and activation of the AKT pathway	Dean et al. (2010)	Dean et al. (2010)	Dean et al. (2010)
		Ribociclib, in combination with GSK2334470(PDK1inhibitor) or alpelisib (PI3Kα inhibitor), decreased xenograft tumor growth more potently than each drug alone			
PI3K/mTOR	HR + BC	Treatment with the p110α-selective PI3K inhibitor, alpelisib (BYL719), completely blocked the progression of acquired CDK4/6 inhibitor-resistant xenografts in the absence of continued CDK4/6 inhibitor treatment in models of both PIK3CA mutant and wild-type ER+/HER2- breast cancer	Condorelli et al. (2018)	Condorelli et al. (2018); Wander et al. (2020)	Condorelli et al. (2018)
FGFR	HR + BC	High FGFR2 expression correlates with sensitivity to CDK4/6i + endocrine therapy; activating mutations and/or amplifications of FGFR1 and FGFR2 in ER + -resistant breast cancer patients	Young et al. (2014); Yoshida et al. (2019)	Yoshida et al. (2019); Young et al. (2014); O'Brien et al. (2020); Chen et al. (2019)	Young et al. (2014)
	ER + MBC	41% patients after progression on CDK4/6 inhibitors identified FGFR1/2 amplification or activating mutations			
	KRAS-mutant Non-Small Cell Lung Cancer	FGFR1–MAP kinase–mTOR pathway resulting in increased expression of D-cyclins and CDK6 that confers Palbociclib resistance			
FAT1-hippo	ER + BC	Loss of the FAT1 Tumor Suppressor Promotes Resistance to CDK4/6 Inhibitors via the Hippo Pathway	Jansen et al. (2017)	Jansen et al. (2017)	Jansen et al. (2017)
WEE1	ER + BC cells	Targeting WEE1 Inhibits Growth of Breast Cancer Cells That Are Resistant to CDK4/6 Inhibitors	Formisano et al. (2019)	Formisano et al. (2019)	Formisano et al. (2019)
miR-432–5	ER + BC cells	Resistance was mediated by exosomal miRNA (miR-432-5p), causing increased expression of CDK6 to overcome G1 arrest and promote cell survival	Mao et al. (2020)	Mao et al. (2020)	Mao et al. (2020)
CDK6	HR + BC	The high expression of CDK6 protein was found to be closely linked with resistance to Palbociclib and Abemaciclib	Croessmann et al. (2019); Pandey et al. (2021)	Croessmann et al. (2019)	
CCNE-CDK2	HR + BC	Over-expression or amplification of CCNE1 and CCNE2 is linked with resistance to treatment in ER+/HER2- breast cancers	Yoshida et al. (2019)	Cen et al. (2012); Gong et al. (2017); Green et al. (2019); Turner et al. (2019); Yoshida et al. (2019); Alves et al. (2021); Maylina et al. (2023)	—
	Gastric Cancer	HR+/HER2- breast cancers that are resistant to treatment have an increased presence of cytoplasmic cyclin E			
		CDK4/6 inhibitors resistance cell due to CCNE1 amplification could be made responsive again by targeting CDK2			
CDKN2A	BC cells	Elevated levels of CDKN2A are indicative of insensitivity to Palbociclib, whereas low levels do not necessarily correlate with sensitivity. It is suggested that high CDKN2 levels may lead to reduced CDK4/6 activity and resistance to CDK4/6 inhibitors. Furthermore, high levels of CDKN2A protein may suggest the loss of RB1 function	Haines et al. (2018)	Herrera-Abreu et al. (2016); Haines et al. (2018); Olmez et al. (2018); Kharenko et al. (2022)	—
	Canine Lymphoma cells				
	Glioblastoma				
	Melanoma				
CCND1	ER + BC	In the case of resistance to Palbociclib and abemaciclib, CCND1 protein levels was found to be significantly upregulated. Genetic mutations that boost cyclin D1 expression were associated with high sensitivity to CDK4/6 inhibitors. Genomic aberrations that activate D-type cyclins were found to be linked with increased sensitivity to the CDK4/6 inhibitor abemaciclib	Sherr (2018); Croessmann et al. (2019)	Croessmann et al. (2019)	—
AKT1	HR + BC	AKT1 amplification and activating mutation were identified in resistant biopsies	Yoshida et al. (2019)	Yoshida et al. (2019)	—
KRAS/HRAS/NRAS	HR + BC	Activating mutations in KRAS, HRAS and amplification in NRAS found in ER+/HER2–metastatic breast cancer with intrinsic and acquired resistance	Yoshida et al. (2019)	Gottesman et al. (2019); Yoshida et al. (2019); Pandey et al. (2022)	—
	KRAS-mutant Non-Small Cell Lung Cancer	FGFR1–MAP kinase–mTOR pathway resulting in increased expression of D-cyclins and CDK6 that confers Palbociclib resistance			
c-Met/TrkA-B	Glioblastoma	Both *in vitro* and *in vivo* that dual inhibition of c-Met/Trk is able to overcome resistance to CDK4/6 inhibition	Guiley et al. (2019)	Guiley et al. (2019)	—
ERBB2	HR + BC	ERBB2 mutations hyperactivate the HER3/PI3K/AKT/mTOR axis	Yoshida et al. (2019)	Yoshida et al. (2019); Pancholi et al. (2020)	—
ER	HR + BC	Inactivation of ER signaling correlates with resistance in breast cancer	O'Leary et al. (2018); Yoshida et al. (2019)	O'Leary et al. (2018); Yoshida et al. (2019)	—
AURKA	HR + BC	AURKA inhibition resulted in prolonged clinical benefit	Yoshida et al. (2019)	Yoshida et al. (2019)	—
CDK4	HR + BC	The phosphorylation of CDK4 at T172 is indicative of its activity and sensitivity to CDK4/6 inhibitors. The amplification of CDK4 is associated with resistance to CDK4/6 inhibitors in sarcoma and glioblastoma	—	Olmez et al. (2018); Pandey et al. (2020)	Guarducci et al. (2018)
	Rhabdomyosarcoma				
	Glioblastoma				
SLC36A1-mTORC1 signaling	Melanoma	Reactivation of mammalian target of rapamycin 1 (mTORC1) signaling through increased expression of the amino acid transporter, solute carrier family 36 member 1 (SLC36A1), drives resistance to CDK4/6 inhibitors	—	El Rassy et al. (2018)	El Rassy et al. (2018)
RON	ESR1 mutant BC	RON was hyperactivated in acquired Palbociclib-resistant (PalbR) models	—	Thangavel et al. (2013)	—
INF	ER + BC	IFN-related signatures were highly enriched in patients with tumors exhibiting intrinsic resistance to CDK4/6i	—	Jin et al. (2020)	—
HDAC5	Prostate and BC	HDAC5 Loss Impairs RB Repression of Pro-Oncogenic Genes and Confers CDK4/6 Inhibitor Resistance	—	Cornell et al. (2019)	—
miR-223	Luminal BC	Anti–miR-223 cells were significantly more resistant to Palbociclib	—	Fallah et al. (2021)	—
miR-106b	MCF-7	miR106b cluster as being efficiently repressed with CDK4/6 inhibition in an E2F and RB-dependent manner	—	Li et al. (2018)	—
TROJAN	ER + BC	The inhibition of TROJAN abolished the activity of CDK2, reversing the resistance to CDK4/6 inhibitor	—	Finn et al. (2020)	—
PLK1	HR + BC	The current study showed the significant association of the pole-like kinase 1 (PLK1) level and Palbociclib resistance. Moreover, the cumulative PLK1 inhibition in the G2/M phase by Abemaciclib proved to be a mechanism of the synergistic effect		De Angelis et al. (2021)	

BC, breast cancer; HR+, hormone receptor-positive; ER+, estrogen receptor positive.

### 4.1 Resistance mechanisms that are shared in Palbociclib, Ribociclib, and Abemaciclib

There are several pathways and mechanisms that have been confirmed to mediate the resistance of Palbociclib, Ribociclib, and Abemaciclib. Loss of RB1 function is a common mechanism of CDK4/6i resistance (O'Leary et al., 2018; Malorni et al., 2016; Herrera-Abreu et al., 2016; Condorelli et al., 2018; Wander et al., 2020; Dean et al., 2010; Young et al., 2014). PI3K inhibitor alpelisib completely blocked the CDK4/6i resistant xenografts progression in both PIK3CA mutant and ER+/HER2-breast cancer (O'Brien et al., 2020; Chen et al., 2019). Kinome-wide siRNA screen identified 3-Phosphoinositide-dependent protein kinase 1 (PDK1) as a key modulator of Ribociclib sensitivity in MCF-7 and exhibited cross-resistance to Palbociclib and Abemaciclib (Jansen et al., 2017). ER + breast cancer cells transduced with Fibroblast growth factor receptor 1 (FGFR1) was resistant to CDK4/6i. Breast cancer cells expressing FGFR1, transduced with FGFR1 or amplified with FGFR1 were resistant to CDK4/6i (O'Leary et al., 2018; Wander et al., 2020; Formisano et al., 2019; Mao et al., 2020; Finn et al., 2020). A loss of FAT1 promotes CDK4/6i resistance via the Hippo Pathway (Li et al., 2018). When WEE1 is targeted, it inhibits the growth of CDK4/6i-resistant breast cancer cells (Fallah et al., 2021). CDK6 expression was increased in response to exosomal miRNAs (miR-432-5p), allowing cells to survive and overcome G1 arrest (Cornell et al., 2019). These pathways and mechanisms are complex and interconnected, and their involvement in mediating resistance to CDK4/6i may vary depending on the specific cancer type and individual patient characteristics. The shared presence of these mechanisms across the three CDK4/6i implies their role as common determinants of resistance to CDK4/6 Inhibition.

### 4.2 Resistance mechanisms may not be shared

Palbociclib, the first CDK4/6i to be approved, has been more thoroughly investigated for its resistance mechanism compared to other CDK4/6i. The following mechanisms were only studied in Palbociclib and not in Abemaciclib and Ribociclib. LncRNA TROJAN bind to NKRF and inhibits the interaction with RELA, restoring CDK2 expression and reversing CDK4/6 resistance (Jin et al., 2020). The miR106b (Thangavel et al., 2013) and miR-223 (Citron et al., 2020) were efficiently repressed with Palbociclib in an E2F and RB-dependent manner. Lack of HDAC5 impairs RB repression of pro-oncogenic genes and confers resistance to Palbociclib (Zhou et al., 2021). Palbociclib-resistant tumors exhibited high levels of IFN-related signatures (De Angelis et al., 2021) and RON hyperactivated (Dustin et al., 2021). The increase of TK1 and CDK9 are associated with clinical resistance to Palbociclib (Del Re et al., 2019). The development of resistance to Palbociclib caused elevated expression of genes including CDK7, the master regulator of the cell cycle. Additionally, loss of ER and RB1 has been shown to increase sensitivity to CDK7 inhibition (Pancholi et al., 2020). Inhibition of PLK1 cumulatively by eribulin or abemaciclib in the G2/M phase proved to be one mechanism for synergistic effects in Palbociclib resistance (Pandey et al., 2022). It is observed that high levels of CDKN1B may predict resistance to Palbociclib (Gottesman et al., 2019; Guiley et al., 2019). Indeed, the resistance mechanisms mentioned above have only been extensively studied in Palbociclib, and their relevance to resistance to Abemaciclib and Ribociclib is still not clear. However, it is worth noting that Palbociclib was the first CDK4/6i to receive FDA approval, and its specific resistance mechanisms may differ from those of Abemaciclib and Ribociclib. Further research is needed to elucidate the resistance mechanisms of all CDK4/6i.

Inactivation of the ER signaling pathway (O'Leary et al., 2018; Wander et al., 2020) and ERBB2 mutations (Croessmann et al., 2019; Wander et al., 2020) is closely associated with Abemaciclib and Palbociclib resistance. The presence of activating mutations in KRAS and HRAS, as well as amplification in NRAS, have been identified in ER+/HER2-metastatic breast cancer cases exhibiting both intrinsic and acquired resistance for Abemaciclib (Wander et al., 2020) and Palbociclib (Haines et al., 2018; Sherr, 2018; Wander et al., 2020). AKT1 amplification and activating mutation were identified in Abemaciclib and Palbociclib resistant biopsies (Wander et al., 2020). Experiments confirmed resistant cells were sensitive to Aurora Kinase (AURKA) inhibition LY3295668 (Wander et al., 2020) and inhibition of c-Met/Trk (Olmez et al., 2018). In the case of resistance to abemaciclib (Gong et al., 2017; Kharenko et al., 2022) and Palbociclib (Kharenko et al., 2022), CCND1 protein levels were found to be significantly upregulated. Genetic mutations that boost cyclin D1 expression were associated with high sensitivity to CDK4/6i. Elevated levels of CDKN2A are indicative of insensitivity whereas low levels do not necessarily correlate with sensitivity. Furthermore, high levels of CDKN2A protein may suggest the loss of RB1 function. It is suggested that high CDKN2 levels may lead to reduced activity and resistance to Abemaciclib (Maylina et al., 2023) and Palbociclib (Cen et al., 2012; Young et al., 2014; Green et al., 2019; Maylina et al., 2023). CDK2 Targeting can be used to reactivate resistance cells to Abemaciclib (Wander et al., 2020) and Palbociclib (Herrera-Abreu et al., 2016; Guarducci et al., 2018; Min et al., 2018; Turner et al., 2019; Pandey et al., 2020; Wander et al., 2020; Pandey et al., 2021) due to CCNE1 amplification. The high expression of CDK6 protein was found to be closely linked with resistance to Palbociclib (Kharenko et al., 2022) and Abemaciclib (Yang et al., 2017; Kharenko et al., 2022), which is not seen in Ribociclib resistant cell lines.

While Abemaciclib and Ribociclib were both approved by the FDA around the same time, the specific mechanisms of Ribociclib resistance may be less well-studied. The phosphorylation of CDK4 at T172 is indicative of its activity and sensitivity to Palbociclib in breast cancer, sarcoma and glioblastoma (Cen et al., 2012; Raspé et al., 2017). CDK4 Amplification Reduces Sensitivity to Ribociclib in fusion-positive rhabdomyosarcoma (Olanich et al., 2015). A reactivation of mammalian target of rapamycin 1 (mTORC1) signaling by increasing expression of solute carrier family 36 member 1 (SLC36A1), contributes Palbociclib and Ribociclib resistance in Melanoma (Yoshida et al., 2019). There is currently no evidence of CDK4 or mTORC1 mediated abemaciclib resistance.

All the mechanisms involved in resistance are mainly studied in breast cancer, but there are also Melanoma, Non-Small Cell Lung Cancer, Rhabdomyosarcoma, Glioblastoma, Gastric Cancer, Canine Lymphoma cells, Prostate, Glioblastoma, Which Remind us that CDK4/6i are being widely studied and applied in various fields.

## 5 Conclusion and perspectives

CDK4/6i will still be primarily used against breast cancer in the next 5 years, and its clinical applications will expand to include a range of tumor types. It is important to note, however, that as use of the drug increases, the incidence of drug resistance increases, and new combination regimens and other targeted drugs may become available as a result. Currently, the resistance mechanisms of the three drugs remain largely unknown, and there are differences in the resistance mechanisms among them, necessitating further exploration and research.
